# Effects of Oral *Myrtus communis* L. Extract on Serum IgG, Growth Hormone, Oxidative Status, and Serum Biochemistry in Weaned Ramlıç Lambs

**DOI:** 10.3390/ani16172761

**Published:** 2026-09-02

**Authors:** Durmuş Fatih Başer, Mehmet Ali Erfidan, Turan Civelek

**Affiliations:** 1 Department of Internal Medicine, Faculty of Veterinary Medicine, Afyon Kocatepe University, 03204 Afyonkarahisar, Turkey; tcivelek@aku.edu.tr; 2 Department of Veterinary Medicine, Emirdağ Vocational School, Afyon Kocatepe University, 03600 Afyonkarahisar, Turkey; maerfidan@aku.edu.tr

**Keywords:** *Myrtus communis*, phytogenic feed additive, humoral immunity, growth hormone, oxidative status, weaned lambs, metabolic homeostasis

## Abstract

This study evaluated the effects of *Myrtus communis* L. extract on growth performance, growth hormone (GH), humoral immune response, oxidative stress indicators, and serum biochemical parameters in weaned Ramlıç lambs. Forty-five clinically healthy male lambs were allocated to three groups: control (*n* = 9), 0.5 mL/kg extract (*n* = 18), and 1 mL/kg extract (*n* = 18). The extract was administered orally once daily for 45 days. Body weight measurements and blood sampling were performed on days 0, 15, 30, and 45. Serum samples were analyzed for GH, IgG, total antioxidant status (TAS), total oxidant status (TOS), and selected biochemical parameters, whereas oxidative stress index (OSI) was calculated from TAS and TOS values. Longitudinal effects of dose, sampling day, and their interaction were evaluated using a linear mixed-effects model. Although body weight increased in all groups, no significant differences were detected among the groups (*p* > 0.05). A significant day × dose interaction was observed for GH (*p* = 0.004). IgG concentrations were significantly higher in the 1 mL/kg group on day 30 and in both extract-treated groups on day 45 than in controls. Extract administration did not cause significant changes in TAS, TOS, OSI, liver enzymes, renal function parameters, glucose, or lipid profile. In conclusion, *M. communis* extract was biochemically well tolerated and was associated with increased serum IgG concentrations but did not exert a marked effect on growth performance or systemic oxidant–antioxidant balance. Further studies under different physiological conditions and with longer administration periods are needed to clarify its mechanisms of action and potential use in ruminant husbandry.

## Simple Summary

Plant-derived feed additives are being explored as natural tools to support animal health. This study evaluated the effects of *Myrtus communis* L. extract in weaned Ramlıç lambs. Forty-five healthy male lambs received no extract, 0.5 mL/kg extract, or 1 mL/kg extract orally once daily for 45 days. The extract did not improve body weight gain or markedly change oxidative balance. However, lambs receiving the extract had higher immunoglobulin G levels at selected time points, suggesting a possible supportive effect on humoral immunity. No adverse changes were observed in the measured liver, kidney, glucose, or lipid-related parameters. These findings indicate that *M. communis* extract may serve as a safe phytogenic feed additive supporting humoral immunity.

## 1. Introduction

In animal production, legal restrictions on the use of antibiotic-based growth promoters, together with increasing consumer expectations for safe and sustainable livestock production, have increased interest in phytogenic feed additives [1,2,3]. Phytogenic feed additives are natural products derived from herbs, spices, plant extracts, and essential oils that contain biologically active secondary metabolites such as flavonoids, tannins, saponins, alkaloids, and terpenoids [4]. These compounds have the potential to optimize digestive functions, immune responses, oxidant–antioxidant balance, growth performance, and metabolic homeostasis in animals [5,6,7].

Postnatal development in young ruminants is a critical period during which several physiological changes occur concurrently, including the transition from milk feeding to solid feed intake, maturation of rumen functions, and exposure to environmental stressors. During this period, growth performance is closely associated with metabolic health and the maintenance of oxidant–antioxidant balance [8]. Growth hormone (GH) directly affects growth rate and feed efficiency by enhancing protein synthesis and promoting lipolysis [9]. Concurrently, immunoglobulin G (IgG) levels are among the key determinants of reduced morbidity and mortality in animals [8,10]. However, rapid growth, dietary transition, and environmental stressors may disrupt oxidative balance and weaken the antioxidant defense system [8,11,12,13]. The antioxidant capacity of plant-derived nutraceuticals may contribute to redirecting metabolic energy toward growth and immune functions rather than cellular repair by limiting oxidative damage [1]. Therefore, PFAs are considered not only as alternatives to antibiotic growth promoters but also as part of a holistic nutritional approach to support animal health and sustainable production [3,14].

Myrtle (*Myrtus communis* L.) is an aromatic plant rich in bioactive compounds, widely distributed in the Mediterranean basin and commonly used in traditional medicine [15,16,17]. The leaves and fruits of myrtle are rich in flavonoids, including quercetin, catechin, and myricetin, as well as tannins and anthocyanins [18,19,20]. The ability of *M. communis* extracts to scavenge free radicals and inhibit lipid peroxidation has been demonstrated in various in vitro studies [21,22,23]. Similarly, myrtle extracts have been reported to remove free radicals and reduce lipid peroxidation [16,24]. Experimental studies have shown that *M. communis* extract significantly increases blood glutathione (GSH) levels and superoxide dismutase (SOD) activity while reducing malondialdehyde (MDA) concentrations [25,26]. However, most of these findings have been obtained from in vitro systems or experimental disease models, and their systemic effects in healthy ruminants have not yet been sufficiently clarified. *Myrtus communis* is rich in flavonoids such as myricetin and other bioactive compounds that may modulate cellular metabolic signaling and influence endocrine mediators, including leptin and adiponectin [7,17]. Such endocrine modulation may, in turn, interact with the somatotropic axis and GH–IGF-1 signaling, which plays a central role in nutrient partitioning, protein synthesis, and somatic growth [3].

When the available studies on the effects of *M. communis* in ruminants are considered, supplementation with myrtle extract in calves has been reported to affect the immune system and improve growth performance [7]. In addition, dietary supplementation with myrtle extract during the transition period in dairy cows has been reported to increase dry matter intake and milk yield [27]. In sheep, the inclusion of myrtle leaves in the diet has been shown to increase protein digestibility and reduce serum glucose and triglyceride concentrations [28]. Similarly, the use of myrtle berries in dairy sheep has been reported to improve ruminal nitrogen balance [29]. Collectively, these studies suggest that *M. communis* may exert beneficial effects on performance, metabolism, and immunity in different ruminant species and physiological stages. However, differences among previous studies in ruminant species, physiological stage, and application protocol limit direct comparisons, and evidence remains scarce regarding the combined evaluation of growth hormone and oxidative balance [7,30]. Moreover, studies evaluating the time- and dose-dependent effects of *M. communis* extract on body weight change, serum GH and IgG concentrations, systemic oxidant–antioxidant balance, and biochemical safety indicators in lambs are limited.

This study was conducted to evaluate the effects of two oral doses of *M. communis* extract (0.5 and 1 mL/kg), administered for 45 days, on body weight change, serum GH and IgG concentrations, total antioxidant status (TAS), total oxidant status (TOS), oxidative stress index (OSI), and selected biochemical parameters in weaned male Ramlıç lambs. Overall, the study aimed to provide an integrated assessment of the effects of *M. communis* extract on growth-related responses, humoral immunity, oxidant–antioxidant balance, and metabolic safety. It was hypothesized that administration of the extract would support the humoral immune response and modulate growth-related hormonal responses without adversely affecting metabolic safety.

## 2. Materials and Methods

### 2.1. Ethical Approval

This study was conducted with the approval of the Afyon Kocatepe University Animal Experiments Local Ethics Committee (AKUHADYEK; approval date: 26 March 2025; approval no. 49533702-314).

### 2.2. Animals

The study was carried out at the Afyon Kocatepe University Education, Research and Application Farm. Ramlıç lambs, raised in a flock of approximately 1000 small ruminants and adapted to regional conditions, were used as the study material.

Male Ramlıç lambs born between January and April of the same year were included in the study. The study was initiated after a 15-day adaptation period following weaning. At the end of the adaptation period, all lambs underwent general clinical and physical examinations. A total of 50 clinically healthy male lambs were enrolled in the study.

At the beginning of the study, the mean ages of the lambs were 113.67 ± 18.11 days in the control group, 116.83 ± 17.24 days in the 0.5 mL/kg group, and 119.33 ± 16.11 days in the 1 mL/kg group. Although some individual variation in age was present, the group mean ages were comparable. After completion of the adaptation period, the lambs were classified according to their initial body weights and then randomly allocated to the experimental groups to achieve comparable initial body weight distributions among groups.

Initially, 50 clinically healthy male Ramlıç lambs were randomly allocated to three experimental groups: control (*n* = 10), 0.5 mL/kg *Myrtus communis* L. extract (*n* = 20), and 1 mL/kg *Myrtus communis* L. extract (*n* = 20). During the experimental period, five lambs were excluded per the predefined exclusion criteria due to health conditions unrelated to the experimental treatments. Specifically, one lamb in the control group developed clinical signs of pneumonia; in the 0.5 mL/kg group, one lamb developed pneumonia and one lamb developed lameness; and in the 1 mL/kg group, one lamb developed pneumonia and one lamb developed lameness. Consequently, the final study groups consisted of the control group (*n* = 9), the 0.5 mL/kg group (*n* = 18), and the 1 mL/kg group (*n* = 18), resulting in a total of 45 lambs included in the statistical analyses. No significant differences in initial body weight were detected among the groups either before (*n* = 50) or after the exclusions (*n* = 45) (ANOVA, *p* > 0.05). Body weight measurements were performed in the morning before feeding on days 0, 15, 30, and 45 using a calibrated electronic scale. Each individual lamb was considered an independent biological replicate; therefore, the reported *n* values represent the number of biological replicates in each group.

### 2.3. Administration Protocol

The study was conducted using a randomized, controlled, parallel-group experimental design. After completion of the adaptation period, the lambs were allocated to the control group and to groups administered 0.5 mL/kg and 1 mL/kg of *Myrtus communis* L. extract. To identify the animals by their extract-administered groups and to prevent administration errors, lambs in each group were fitted with differently colored neck collars.

Lambs in the extract-administered groups received *M. communis* extract orally once daily for 45 days at doses of 0.5 or 1 mL/kg body weight. No extract was administered to the control group. The selected dose levels were based on a previously published study evaluating oral *Myrtus communis* L. extract supplementation in Holstein calves [7]. The daily extract dose to be administered was recalculated based on individual body weights measured on days 0, 15, and 30; body weight on day 45 was recorded for end-of-study evaluation. Thus, the 0.5 mL/kg body weight and 1 mL/kg body weight doses were maintained throughout the study. Updated dose amounts were used for subsequent administrations after each body weight measurement. All administrations were performed at the same time each day. During the study, the lambs were clinically observed daily, and their general health status was monitored.

Body weight measurements and blood sampling were performed on days 0, 15, 30, and 45 of the study. Serum samples were analyzed for growth hormone (GH), immunoglobulin G (IgG), total antioxidant status (TAS), total oxidant status (TOS), and selected serum biochemical parameters. The oxidative stress index (OSI) was calculated using TAS and TOS results.

### 2.4. Housing and Feeding Conditions

During the study, all groups were housed together in the same housing area and were subjected to the same management, feeding, and flock management practices; thus, potential confounding effects arising from environmental factors were minimized. Animals had ad libitum access to clean water.

Alfalfa hay was provided ad libitum. Concentrate feed was offered to groups at a rate of 1.1 kg per lamb per day, divided into two meals. Individual feed intake was not recorded. The concentrate ration was formulated to contain corn (40.5%), barley (30.1%), soybean meal (15.0%), sunflower meal (7.0%), wheat bran (4.0%), limestone (1.5%), dicalcium phosphate (DCP, 1.2%), salt (0.5%), and a vitamin–mineral premix (0.2%). All animals were fed the same ration throughout the study, and no changes were made to the feeding program.

### 2.5. Procurement and Analysis of Myrtus communis L. Plant Extract

*Myrtus communis* L. extract was obtained from a commercial manufacturer (Biyoderm^®^, ArsArthro Biyoteknoloji A.Ş., Ankara, Turkey). Information regarding the preparation of the extract was provided by the manufacturer. Accordingly, 2 g of dried *M. communis* material, consisting of a leaf and root mixture, was mechanically homogenized and then subjected to aqueous extraction in sterile distilled water adjusted to pH 4.5 at 98 °C for 22 min. After extraction, the mixture was cooled to room temperature, the pH was readjusted to 4.5 as needed, and the extract was clarified by filtration through a 0.2 mm pore-size filter. The resulting filtrate was transferred into bottles and stored at 4 °C until use. All extract used throughout the 45-day experimental period was obtained from the same production batch and stored at 4 °C under the recommended storage conditions until administration.

Phytochemical characterization of the extract was performed to determine its phenolic constituents using liquid chromatography–quadrupole time-of-flight mass spectrometry (LC-QTOF-MS; Agilent Technologies, Santa Clara, CA, USA) and Orbitrap high-resolution mass spectrometry (Thermo Electron, Bremen, Germany). Chromatographic separation was carried out using an Agilent Poroshell 120 EC-C18 column (3.0 × 100 mm, 2.7 µm). Mobile phase A consisted of water containing 10 mM ammonium formate and 0.1% formic acid, whereas mobile phase B consisted of methanol containing 0.1% formic acid. The mass spectrometer ion source conditions were set as follows: drying gas temperature, 325 °C; gas flow rate, 10 L/min; and nebulizer pressure, 45 psig.

The amino acid profile of the extract was analyzed using high-performance liquid chromatography (HPLC; Shimadzu, Columbia, MD, USA), and its mineral composition was determined using inductively coupled plasma mass spectrometry (ICP-MS; Shimadzu, Columbia, MD, USA). The amino acid, phenolic compound, and mineral composition of the extract are presented in Table 1. The measured concentrations of Pb, Cd, Hg, and As in the extract were below the maximum levels established for the relevant animal-feed categories under EU Directive 2002/32/EC.

### 2.6. Blood Sample Collection

Blood samples were collected from all lambs on days 0, 15, 30, and 45 of the study in the morning, before feeding, while the animals were fasted. Samples were collected aseptically from the jugular vein into 8 mL vacuum tubes containing a gel separator and clot activator. Approximately 8 mL of blood was collected from each lamb at each sampling time, and this volume remained within the institutional animal welfare limits for repeated blood sampling in growing lambs.

After allowing the blood samples to clot at room temperature, serum was separated by centrifugation at approximately 2180× *g* for 15 min using a refrigerated centrifuge (M 4815 PR, Elektro-mag, İstanbul, Türkiye). The obtained serum samples were transferred into Eppendorf tubes and stored at −20 °C until analysis. To minimize biological variation associated with circadian rhythm, all blood samples were collected in the morning and within the same time interval across all groups on each sampling day.

### 2.7. Serum Biochemical Analyses

Alanine aminotransferase (ALT), aspartate aminotransferase (AST), alkaline phosphatase (ALP), gamma-glutamyl transferase (GGT), glucose, blood urea nitrogen (BUN), urea, creatinine, total cholesterol, triglyceride (TG), high-density lipoprotein cholesterol (HDL), low-density lipoprotein cholesterol (LDL), and total protein (TP) concentrations were analyzed in serum samples.

Serum biochemical analyses were performed using the Cobas^®^ 8000 modular analyzer series, c703 module (Roche Diagnostics GmbH, Mannheim, Germany; Hitachi High-Technologies Corporation, Tokyo, Japan), in accordance with the manufacturer’s recommended commercial reagents, calibration procedures, and internal quality control protocols. All samples were analyzed in the same laboratory using the same analytical system and under identical analytical conditions.

The estimated glomerular filtration rate (eGFR) was automatically calculated from serum creatinine concentrations using the Modification of Diet in Renal Disease (MDRD) equation, which was incorporated into the analyzer software. Because this equation was originally developed for human clinical use and has not been validated specifically in sheep, the resulting eGFR values were interpreted as approximate rather than as validated measurements of ovine glomerular filtration rate.

### 2.8. Oxidant–Antioxidant and ELISA Analyses

Serum TAS and TOS levels were determined using commercial colorimetric assay kits in accordance with the manufacturer’s instructions (Elabscience Biotechnology Co., Ltd., Wuhan, Hubei, China). Serum IgG and GH concentrations were analyzed using species-specific commercial ELISA kits. FineTest^®^ Sheep IgG ELISA Kit and FineTest^®^ Sheep GH ELISA Kit (Wuhan Fine Biotech Co., Ltd., Wuhan, Hubei, China) were used for IgG and GH analyses, respectively.

Absorbance measurements for TAS, TOS, IgG, and GH analyses were performed using a Thermo Scientific Multiskan™ FC Microplate Photometer (Thermo Fisher Scientific, Vantaa, Finland). All analyses were conducted according to the manufacturers’ protocols, and sample concentrations were calculated using standard curves. Results were expressed as mmol Trolox equivalent/L for TAS, µmol H_2_O_2_ equivalent/L for TOS, mg/mL for IgG, and pg/mL for GH.

### 2.9. Calculation of the Oxidative Stress Index

The oxidative stress index (OSI) was calculated based on the ratio of TOS to TAS. Before calculation, TAS values were converted from mmol Trolox equivalent/L to µmol Trolox equivalent/L. OSI was then calculated using the following formula [31,32]:

OSI (arbitrary units) = [TOS (µmol H_2_O_2_ equivalent/L)/TAS (µmol Trolox equivalent/L)] × 100

### 2.10. Statistical Analysis

All statistical analyses were performed using Minitab 18 software. In the study, ELISA data and biochemical parameters were evaluated by dose group and measurement day. The dose groups consisted of three levels: control, 0.5 mL/kg, and 1 mL/kg; the measurement days consisted of four levels: days 0, 15, 30, and 45. One-way analysis of variance (ANOVA) was used to evaluate differences among dose groups separately for each measurement day. In these analyses, the dose group was considered as a fixed factor, and Tukey’s multiple comparison test was used to determine differences among group means. In addition, a linear mixed-effects model was used to evaluate the effects of dose group, measurement day, and the dose × day interaction. Dose group, measurement day, and their interaction were included as fixed effects, whereas the individual lamb was included as a random effect to account for repeated measurements within animals. Variance components were estimated using restricted maximum likelihood (REML), and the Kenward–Roger approximation was used for the denominator degrees of freedom. The *p*-values for the dose × day interaction obtained from the linear mixed-effects model were presented in the tables as *p*Dose × Day. Results were presented as mean ± standard deviation in the tables. Means indicated by different lowercase letters represent differences among dose groups within the same sampling day, whereas means indicated by different uppercase letters represent differences among sampling days within the same dose group (*p* < 0.05). A significance level of *p* < 0.05 was accepted for all statistical evaluations.

## 3. Results

### 3.1. Animal Material and Growth Performance

The study was conducted with a total of 45 male Ramlıç lambs, after excluding 5 lambs based on the predefined exclusion criteria. At the beginning of the study, no difference in body weight was detected among the groups (*p* > 0.05). Body weight increased progressively over time in all groups; however, no differences in body weight were detected among the control, 0.5 mL/kg, and 1 mL/kg groups at any measurement day (*p* > 0.05). Over the 45-day experimental period, average daily gain was 0.29 ± 0.06, 0.33 ± 0.05, and 0.31 ± 0.04 kg/day in the control, 0.5 mL/kg, and 1 mL/kg groups, respectively. Corresponding total weight gains were 12.89 ± 2.82, 14.79 ± 2.03, and 13.86 ± 1.96 kg, while percentage weight gains were 31.79 ± 7.70%, 38.14 ± 8.93%, and 35.99 ± 13.06%, respectively (Table 2).

### 3.2. Growth Hormone and Humoral Immune Response

**Figure 1:**
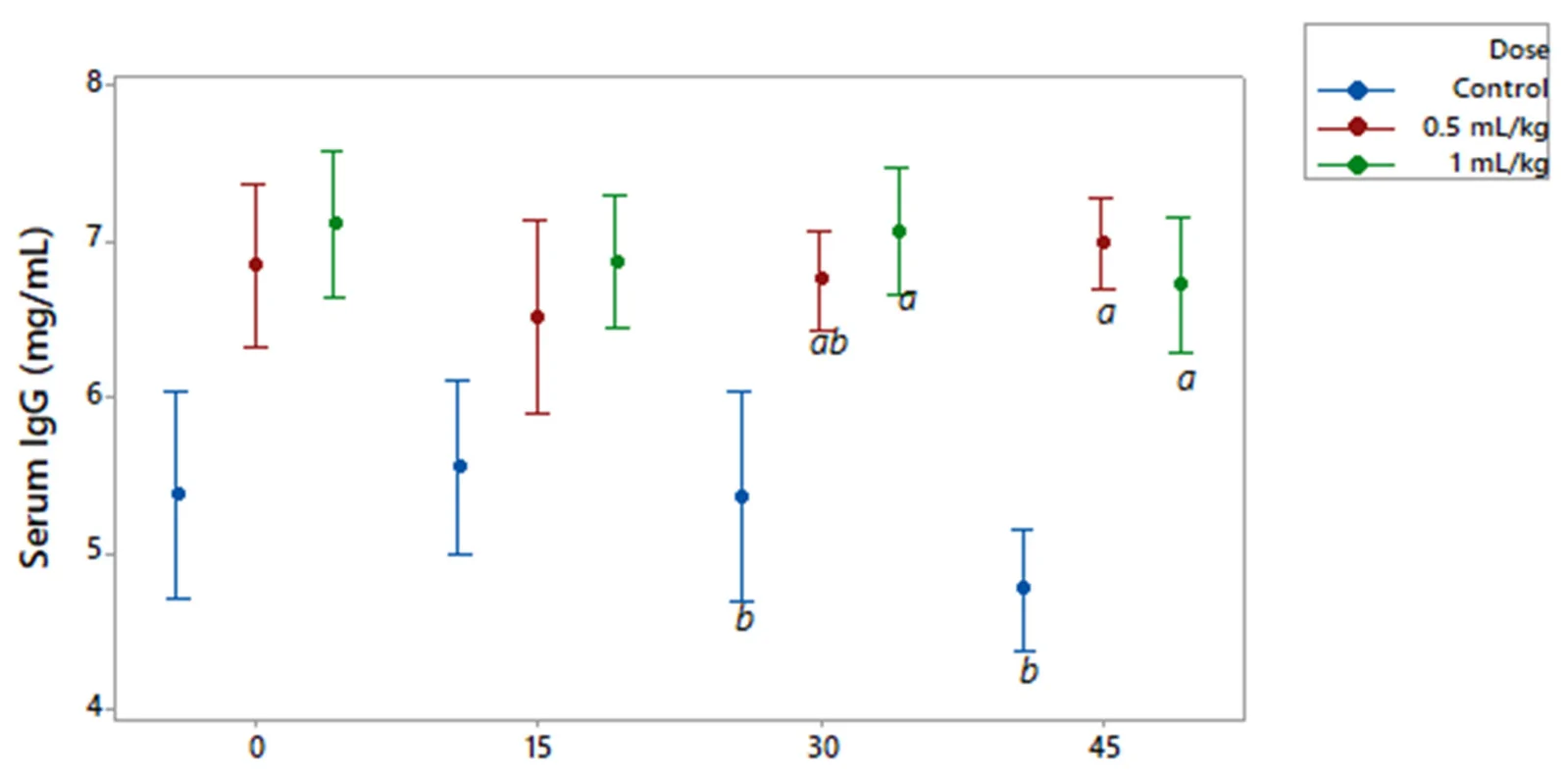
Changes in serum IgG concentrations over the study period in the control, 0.5 mL/kg *Myrtus communis* L. extract, and 1 mL/kg *Myrtus communis* L. extract groups. Data were presented as mean ± SD. Each individual lamb was considered an independent biological replicate; *n* = 9, 18, and 18 for the control, 0.5 mL/kg, and 1 mL/kg groups, respectively. Different lowercase letters indicate differences among treatment groups within the same sampling day (*p* < 0.05). IgG, immunoglobulin G; SD, standard deviation.

No day × dose interaction was detected for serum IgG concentrations (*p*Dose × Day = 0.921). However, on day 30, serum IgG concentration was higher in the 1 mL/kg group than in the control group (*p* < 0.05). On day 45, IgG concentrations were higher in both extract-administered groups (0.5 and 1 mL/kg) than in the control group (*p* < 0.05) (Figure 1, Table 3).

For serum GH concentrations, a day × dose interaction was detected (*p*Dose × Day = 0.004). Between-group differences were observed only on days 0 and 15, whereas no differences were detected on days 30 and 45. In particular, on day 15, GH concentration was higher in the 1 mL/kg group than in the 0.5 mL/kg group (*p* < 0.05) (Table 3).

### 3.3. Oxidative Stress Parameters

No day × dose interaction was observed for serum TAS, TOS, or OSI values. Similarly, no between-group differences were detected in TAS, TOS, or OSI at any measurement day (*p* > 0.05). However, time-dependent within-group changes in TOS and OSI were observed only in the 0.5 mL/kg group (Table 3).

### 3.4. Renal Function Parameters and Glucose

No differences were detected among the groups in serum BUN, urea, creatinine, or eGFR values on any measurement day (*p* > 0.05). In addition, no day × dose interaction was detected for these parameters. However, BUN and urea levels showed time-dependent changes in both extract-administered groups (0.5 and 1 mL/kg) (*p* < 0.001) (Table 4).

Serum glucose levels decreased over time in all groups during the study (*p* < 0.001). However, no between-group differences or day × dose interaction were detected on any measurement day (Table 4).

### 3.5. Liver Enzymes and Total Protein

No differences were detected among the groups in serum ALP, AST, GGT, or total protein levels on any measurement day, and no day × dose interaction was detected for these parameters. However, ALP levels decreased over time in both extract-administered groups, whereas AST levels increased over time only in the 0.5 mL/kg group (*p* < 0.05) (Table 5). Between-group differences in serum ALT levels were detected on days 0 and 15 (*p* < 0.05), whereas no differences were found among the groups on days 30 and 45. In addition, ALT levels increased over time in the 0.5 mL/kg group (*p* < 0.001). However, no day × dose interaction was detected for ALT (Table 5).

### 3.6. Lipid Profile

No differences were detected among the groups in serum total cholesterol, HDL, or LDL levels on any measurement day, and no day × dose interaction was detected for these parameters (Table 6).

Serum triglyceride levels showed time-dependent changes in all groups during the study (*p* < 0.05). A between-group difference was detected only on day 15 (*p* = 0.021), whereas no differences were found among the groups on the other measurement days. In addition, no day × dose interaction was detected for triglyceride levels (Table 6).

## 4. Discussion

In this study, the time- and dose-dependent effects of *Myrtus communis* L. extract, an aromatic plant native to the Mediterranean basin, on growth performance, humoral immune response, oxidative balance, and metabolic homeostasis were evaluated in lambs. The findings indicate that *M. communis* extract did not adversely affect liver or renal function indicators at the administered doses and was biochemically well tolerated. The higher serum IgG concentrations observed at selected sampling points suggest an association between extract administration and serum IgG levels. In contrast, the absence of significant differences among groups in TAS, TOS, and OSI values indicates that the extract did not markedly affect systemic oxidant–antioxidant balance in healthy lambs. Moreover, extract administration did not provide a clear advantage over the control group in terms of body weight gain. This finding suggests that the observed immunological changes may not have been reflected in growth performance within the short-term study period. The distinctive aspect of this study is that it evaluated growth hormone, humoral immune response, oxidative stress parameters, and biochemical safety indicators of *M. communis* extract together within the same experimental model in lambs.

When serum IgG levels were evaluated, the group administered the 1 mL/kg extract showed significantly higher levels than the control group on day 30. On day 45, IgG levels were higher in both extract groups (1 and 0.5 mL/kg) than in the control group (Table 3; Figure 1). These findings suggest that *M. communis* extract may be associated with higher serum IgG concentrations in lambs at later sampling points; however, the dose × day interaction was not significant, and the extract groups showed numerically higher IgG concentrations at baseline. This effect may be associated with the immunomodulatory properties of flavonoids, particularly myricetin, which was present at a high concentration in the extract [7]. Flavonoids and other polyphenolic compounds have been reported to play roles in immune cell activation, cytokine synthesis, and macrophage regulation [33]. In addition to myricetin, other phenolic constituents identified in the extract, including catechin and quercetin, may also have contributed to the observed biological response. These flavonoids have been associated with immunomodulatory activity, while the phenolic constituents of *M. communis* have also been linked to free-radical scavenging, inhibition of lipid peroxidation, and enhancement of antioxidant defense mechanisms [16,21,22,23,24,25,26,33]. Therefore, the phenolic profile of the extract may be associated with the observed increase in serum IgG concentrations. However, because the individual effects of these compounds and the underlying cellular mechanisms were not directly evaluated in the present study, and the proposed immunomodulatory mechanisms are largely based on evidence from other species or related bioactive compounds, this interpretation should be considered a plausible mechanistic explanation rather than a demonstrated causal relationship. However, the absence of significant changes in oxidative stress markers in the present study suggests that the observed increase in IgG cannot be explained solely by antioxidant mechanisms. Therefore, it should not be overlooked that the immunomodulatory effect may have occurred through different cellular and molecular mechanisms [3,14]. Environmental and nutritional stress can impair immune responses in young ruminants [8], whereas appropriate nutritional interventions may support immune functions [13]. In addition, phytogenic additives have been reported to modulate systemic immune responses through the gastrointestinal microbiota and gut-associated lymphoid tissue (GALT) [34]; however, these mechanisms were not directly evaluated in the present study.

The present results are consistent with a study reporting that myrtle leaf and root extracts increased serum IgG concentrations in Holstein calves [7]. The beneficial effects of plant bioactives on humoral immunity in ruminants are further supported by findings showing that supplementation with banana and/or garlic leaves increased serum IgG1 and IgG2 levels in sheep [5]. Similarly, antioxidant supplementation in pregnant ewes has been reported to improve passive transfer of immunity in offspring [10]. In poultry studies, myrtle oil has also been reported to support antibody response [35] and to reduce aflatoxin-induced immunosuppression [36]. Taken together, these studies suggest that similar associations between *M. communis* supplementation and immune-related outcomes have been reported across different animal species.

The increase in IgG observed in this study suggests that *M. communis* extract may be associated with higher serum IgG concentrations in healthy weaned lambs. However, longer-term studies conducted under different physiological conditions are needed to determine whether this immunological effect is reflected in clinical resistance to infections, vaccine response, or long-term production performance.

When findings on growth hormone (GH) and growth performance were evaluated together, a significant day × dose interaction was detected for GH levels (*p*D × G = 0.004). In particular, on day 15, the GH level in the 1 mL/kg dose group was higher than that in the 0.5 mL/kg group (Table 3). Body weight increased over time in all groups during the study; however, no statistically significant difference was detected between the control and *M. communis* extract-administered groups (Table 2). GH is one of the key hormonal regulators of postnatal growth by stimulating protein synthesis, enhancing lipolysis, and regulating nutrient partitioning among tissues. This process, mediated by the somatotropic axis, plays an important role in regulating growth rate and feed efficiency [9].

In the present study, the fact that the early increase in GH observed in the high-dose group did not translate into a body weight advantage at the end of the study indicates that the early hormonal response was not reflected in somatic growth within the short term. This suggests that a transient increase in GH levels alone does not determine growth performance and that growth is influenced by multiple factors, including energy balance, nutrient supply, and physiological status [9,37].

Studies have reported that bioactive compounds derived from aromatic plants may support anabolic processes by influencing the somatotropic axis [38,39]; however, this effect may vary with the duration of administration, the energy and protein density of the diet, and the animal’s physiological status [40,41]. Therefore, it is not unexpected that the transient increase in GH observed in the present study did not result in a sustained growth advantage. Our findings indicate that *M. communis* extract may influence the somatotropic axis in lambs during the early period, but that this effect was not reflected in body weight gain under the present study conditions.

The present findings regarding body weight partially contrast with studies reporting that *M. communis* extract or oil increased body weight in Holstein calves [7], broilers [35], and quails at low doses [42]. In contrast, our results are consistent with a study reporting that dietary inclusion of myrtle leaves did not affect body weight change in Arabi sheep [28]. Differences among studies may be attributable to variations in animal species, initial age, dietary composition, extract dose, administration period, and rearing conditions. In addition, the effects of phytogenic additives on growth performance have been reported to be more pronounced, particularly in animals exposed to environmental or metabolic stress [13,36]. Therefore, the absence of a marked effect on growth performance in healthy lambs maintained under appropriate rearing conditions suggests that the response to *M. communis* extract may vary with the animal’s physiological status and environmental conditions.

No significant dose effect of *M. communis* extract administration was detected on serum liver enzymes or renal function indicators in lambs (Table 5). These findings indicate that, at the administered doses, the extract did not exert adverse effects on liver or renal function and was biochemically safe. In the present study, a time-dependent decrease in ALP levels and an increase in ALT levels were observed in the 0.5 mL/kg group. However, the occurrence of similar changes in the control group, the absence of a significant day × dose interaction, and the fact that all values remained within reference ranges suggest that these changes may reflect physiological or metabolic adaptation rather than an effect of extract administration [8,43]. Similarly, the time-dependent changes observed in BUN and urea levels occurred independently of dose and therefore cannot be attributed to extract administration. The present results are consistent with a study reporting that myrtle plant by-products did not affect liver or renal function indicators in lactating ewes [30]. In addition, different phytogenic additives, such as mint and thyme, have been reported not to cause significant changes in liver enzymes (ALT and AST) or creatinine levels in lambs [40]. This suggests that *M. communis* and other phytogenic additives may preserve biochemical homeostasis in healthy animals, whereas their effects may become more evident, particularly in experimental models involving oxidative stress or metabolic disorders [25,36,44].

Serum glucose levels decreased over time in all groups throughout the study (*p* < 0.001) (Table 4). This decrease likely reflects a physiological adaptation independent of extract administration, associated with post-weaning rumen development and the metabolic shift toward greater utilization of volatile fatty acids [8,13]. Although *M. communis* extract has been reported to exert hypoglycemic effects in experimental rat and rabbit models [45,46], no additional decrease in glucose was observed in the extract-treated groups compared with the control group. This discrepancy may be related to differences in species, physiological status, and metabolic conditions. Together with the unchanged liver and renal function indicators, these findings suggest that the extract did not adversely affect glucose homeostasis and was biochemically well tolerated under the present experimental conditions [7,30].

Administration of *M. communis* extract did not produce a significant dose or day × dose effect on TAS, TOS, or OSI in lambs (Table 3). The similar courses of these parameters in the extract-administered and control groups indicate that the extract did not induce a marked change in the systemic oxidant–antioxidant balance at the investigated doses.

The present results are consistent with a study reporting that oxidant–antioxidant parameters, including GPx, MDA, and SOD, showed similar patterns among groups in lactating ewes fed processed myrtle berries [47]. In contrast, our findings partially differ from studies reporting that myrtle extract and myrtle oil increased antioxidant enzyme activities and reduced lipid peroxidation [25,48,49,50]. The main reason for this discrepancy may be differences among animal species, experimental models, and, in particular, the level of oxidative stress under the study conditions. The antioxidant effects of myrtle have been reported to be more evident mostly in models characterized by increased oxidative damage, such as diabetes, burns, or chemical toxicity [25,48,51,52]. In contrast, the use of healthy, clinically normal lambs in the present study may have limited the phytogenic supplement’s ability to induce an additional antioxidant response, as basal antioxidant defense mechanisms were likely functioning adequately. Indeed, it has been reported that the antioxidant effects of bay laurel and myrtle extracts may become more pronounced in disease models associated with oxidative stress [53]. Therefore, the absence of significant changes in TAS, TOS, and OSI parameters in the present study does not necessarily indicate a lack of antioxidant potential of the extract; rather, it suggests that an oxidative burden sufficient to reveal this potential may not have occurred in healthy animals. Consequently, *M. communis* extract did not alter systemic oxidant–antioxidant balance in healthy lambs at the administered doses. Nevertheless, the absence of any increase in TOS and OSI values compared with the control group indicates that the extract did not exert a pro-oxidative effect under the present administration conditions.

This study has some limitations. First, the experimental period was limited to 45 days, which may have restricted a full evaluation of the long-term effects of *M. communis* extract. In addition, because the study was conducted in clinically healthy lambs, the findings cannot be directly generalized to animals under different physiological or pathological conditions. The smaller control group may have reduced statistical power for some control-versus-treatment comparisons and should be considered when interpreting non-significant findings. Because concentrate feed was provided on a group basis and individual feed intake was not measured, potential differences in nutrient intake among individual lambs could not be accounted for when interpreting growth-related outcomes. Furthermore, the control group did not receive a sham oral administration of water or physiological saline; therefore, a potential effect of handling and restraint associated with daily oral administration in the treatment groups cannot be completely excluded. Finally, the mechanisms underlying the observed immunological and hormonal responses were not directly investigated. Future studies conducted under different physiological conditions and supported by more comprehensive molecular analyses may contribute to a better understanding of the mechanisms of action of the extract.

## 5. Conclusions

In conclusion, oral administration of *Myrtus communis* L. extract to weaned Ramlıç lambs for 45 days did not adversely affect liver and renal function indicators or metabolic parameters at the administered doses and was biochemically well tolerated. Extract administration was associated with higher serum IgG concentrations at selected later sampling points; however, it did not induce a marked change in body weight gain or systemic oxidant–antioxidant balance. These findings indicate that *M. communis* extract was biochemically well tolerated and may be associated with favorable changes in serum IgG concentrations in healthy lambs. However, further studies conducted under different physiological conditions, stress models, and longer administration periods will contribute to a more comprehensive understanding of the extract’s mechanisms of action and practical application potential.

## Figures and Tables

**Table 1:** Amino acid profile, phenolic compound content, and elemental composition of *Myrtus communis* L. extract.

**Amino Acid Profile**		**Phenolic Compound Content**	
**Amino Acids**	**Concentration (mg/L)**	**Phenolic Compounds**	**Concentration (mg/L)**
Proline	16,254.60	Myricetin	15.34
Glutamic acid	1767.92	Catechin	4.80
Histidine	1133.84	Quercetin	0.19
Tyrosine	1003.04	Gallic acid	0.13
Tryptophan	907.13	Salicylic acid	0.06
Alanine	691.27	Rosmarinic acid	0.01
Aspartic acid	668.01	**Elemental Composition**	
Arginine	634.54	**Elements**	**Concentration (mg/L)**
Glycine	417.49	Sodium	324.000
Isoleucine	414.90	Magnesium	27.000
Cystine	218.82	Aluminum	1.35
Methionine	281.53	Iron	7.10
Serine	182.91	Tin	1.00
Leucine	173.50	Lead	0.007
Valine	151.64	Cadmium	0.002
		Mercury	0.03
		Arsenic	0.003

**Table 2:** Body weight and growth performance of lambs during the 45-day experimental period.

**Day**	**Group**	***n***	**Body Weight (kg)**	**Minimum**	**Maximum**
0	Control	9	41.84 ± 10.97	25.00	53.80
	0.5 mL/kg	18	40.48 ± 8.95	23.80	55.20
	1 mL/kg	18	41.34 ± 9.00	26.40	52.80
15	Control	9	45.93 ± 11.96	26.20	60.00
	0.5 mL/kg	18	45.40 ± 9.63	26.60	59.60
	1 mL/kg	18	46.11 ± 9.07	30.40	58.40
30	Control	9	50.98 ± 13.15	29.20	67.40
	0.5 mL/kg	18	50.52 ± 9.48	32.00	65.20
	1 mL/kg	18	50.63 ± 8.22	36.80	61.00
45	Control	9	54.73 ± 13.03	32.00	70.80
	0.5 mL/kg	18	55.27 ± 9.47	36.40	69.40
	1 mL/kg	18	55.20 ± 8.00	40.60	66.40
**Trait**	**Group**	***n***	**Mean ± SD**	**Minimum**	**Maximum**
ADG, 0–45 d (kg/day)	Control	9	0.29 ± 0.06	0.16	0.38
	0.5 mL/kg	18	0.33 ± 0.05	0.26	0.42
	1 mL/kg	18	0.31 ± 0.04	0.24	0.41
Total weight gain, 0–45 d (kg)	Control	9	12.89 ± 2.82	7.00	17.00
	0.5 mL/kg	18	14.79 ± 2.03	11.60	19.00
	1 mL/kg	18	13.86 ± 1.96	10.80	18.40
Weight gain, 0–45 d (%)	Control	9	31.79 ± 7.70	22.78	50.00
	0.5 mL/kg	18	38.14 ± 8.93	24.71	52.94
	1 mL/kg	18	35.99 ± 13.06	21.18	69.70

Data are presented as mean ± standard deviation (SD), together with minimum and maximum values. ADG, average daily gain. Total weight gain was calculated as the difference between body weight on Day 45 and Day 0. Percentage weight gain was calculated as [(Day 45 body weight − Day 0 body weight)/Day 0 body weight] × 100.

**Table 3:** Serum GH, IgG, TAS, TOS, and OSI values.

Parameter	Dose	Day 0	Day 15	Day 30	Day 45	*p*_DAY_
GH (pg/mL)	Control	3169 ± 1107 ^a^	2509 ± 607 ^ab^	3104 ± 1211	2247 ± 451	0.102
	0.5 mL/kg	2347 ± 749 ^b^	2039 ± 467 ^b^	2469 ± 758	2305 ± 565	0.245
	1 mL/kg	2517 ± 571 ^ab^	2735 ± 642 ^a^	2412 ± 576	2276 ± 692	0.167
	*p*_dose_	0.038	0.003	0.097	0.972	*p*_DxG_ = 0.004
IgG (mg/mL)	Control	5.37 ± 1.99	5.55 ± 1.69	5.36 ± 2.02 ^b^	4.77 ± 1.17 ^b^	0.794
	0.5 mL/kg	6.83 ± 2.21	6.51 ± 2.65	6.75 ± 1.35 ^ab^	6.98 ± 1.26 ^a^	0.908
	1 mL/kg	7.1 ± 1.98	6.87 ± 1.83	7.06 ± 1.71 ^a^	6.72 ± 1.86 ^a^	0.916
	*p*_dose_	0.123	0.339	0.045	0.002	*p*_DxG_ = 0.921
TOS (µmol H_2_O_2_ equiv./L)	Control	91.33 ± 12.52	71 ± 37.3	89.65 ± 3.66	87.53 ± 4.17	0.132
	0.5 mL/kg	71.09 ± 33.17 ^AB^	67.85 ± 38.09 ^B^	87.83 ± 5.03 ^A^	87.96 ± 5.59 ^A^	0.030
	1 mL/kg	83.8 ± 23.55	66.57 ± 35	81.1 ± 15.85	86.32 ± 11.43	0.059
	*p*_dose_	0.144	0.958	0.084	0.833	*p*_DxG_ = 0.591
TAS (mmol Trolox equiv./L)	Control	1.77 ± 0.48	2.07 ± 0.91	2.22 ± 0.71	1.75 ± 0.16	0.326
	0.5 mL/kg	1.9 ± 0.23	2.22 ± 0.77	1.92 ± 0.33	1.92 ± 0.44	0.161
	1 mL/kg	1.79 ± 0.5	2 ± 0.81	2.4 ± 1.08	2.01 ± 0.44	0.117
	*p*_dose_	0.650	0.728	0.191	0.279	*p*_DxG_ = 0.294
OSI (AU)	Control	5.95 ± 3.71	4.15 ± 2.68	4.37 ± 1.15	5.07 ± 0.68	0.389
	0.5 mL/kg	3.79 ± 1.79 ^AB^	3.28 ± 2.1 ^B^	4.68 ± 0.68 ^A^	4.72 ± 0.71 ^A^	0.009
	1 mL/kg	10.16 ± 24.39	4.15 ± 3.79	3.87 ± 1.5	4.43 ± 0.92	0.368
	*p*_dose_	0.473	0.634	0.122	0.156	*p*_DxG_ = 0.570

Data were presented as mean ± SD. The experimental groups were the control group, the 0.5 mL/kg *Myrtus communis* L. extract, and the 1 mL/kg *Myrtus communis* L. extract. Each individual lamb was considered an independent biological replicate; *n* = 9, 18, and 18 for the control, 0.5 mL/kg, and 1 mL/kg groups, respectively. Different lowercase letters indicate differences among treatment groups within the same sampling day, whereas different uppercase letters indicate differences among sampling days within the same treatment group (*p* < 0.05). *p*Day, effect of sampling day; *p*Dose, effect of dose group; *p*Dose × Day, dose × day interaction. GH, growth hormone; IgG, immunoglobulin G; TAS, total antioxidant status; TOS, total oxidant status; OSI, oxidative stress index; SD, standard deviation.

**Table 4:** Serum BUN, urea, creatinine, eGFR, and glucose values.

Parameter	Dose	Day 0	Day 15	Day 30	Day 45	*p*_DAY_
BUN (mg/dL)	Control	17.26 ± 4.46	20.87 ± 5.56	19.68 ± 3.35	17.18 ± 4.52	0.243
	0.5 mL/kg	15.02 ± 3.78 ^C^	21.69 ± 5.19 ^A^	18.59 ± 2.61 ^AB^	17.20 ± 2.37 ^BC^	0.000
	1 mL/kg	15.77 ± 3.36 ^B^	21.97 ± 5.42 ^A^	20.02 ± 2.25 ^A^	19.38 ± 3.44 ^A^	0.000
	*p*_dose_	0.356	0.880	0.260	0.107	*p*_DxG_ = 0.433
Urea (mg/dL)	Control	36.93 ± 9.5	44.66 ± 11.91	42.09 ± 7.13	36.78 ± 9.69	0.244
	0.5 mL/kg	32.16 ± 8.09 ^C^	46.42 ± 11.12 ^A^	39.79 ± 5.59 ^AB^	36.78 ± 5.06 ^BC^	0.000
	1 mL/kg	33.77 ± 7.2 ^B^	47.01 ± 11.59 ^A^	42.83 ± 4.85 ^A^	41.51 ± 7.38 ^A^	0.000
	*p*_dose_	0.356	0.880	0.265	0.103	*p*_DxG_ = 0.429
Creatinine (mg/dL)	Control	0.61 ± 0.13	0.68 ± 0.11	0.62 ± 0.07	0.72 ± 0.12	0.124
	0.5 mL/kg	0.58 ± 0.12	0.66 ± 0.12	0.62 ± 0.12	0.66 ± 0.13	0.163
	1 mL/kg	0.64 ± 0.14 ^B^	0.69 ± 0.12 ^AB^	0.65 ± 0.12 ^AB^	0.75 ± 0.09 ^A^	0.025
	*p*_dose_	0.376	0.711	0.696	0.071	*p*_DxG_ = 0.335
eGFR	Control	116.44 ± 11.56	110.67 ± 6.78	114.22 ± 5.61	107.78 ± 7.55	0.138
	0.5 mL/kg	118.94 ± 11.51	112.5 ± 9.69	114.94 ± 8.92	112.06 ± 9.1	0.147
	1 mL/kg	114.28 ± 12.29	110 ± 8.13	112.83 ± 9.62	106.72 ± 5.56	0.080
	*p*_dose_	0.503	0.673	0.765	0.102	*p*_DxG_ = 0.781
Glucose (mg/dL)	Control	81.22 ± 13.56 ^A^	65.67 ± 9.49 ^B^	63.89 ± 9.94 ^B^	60.44 ± 9.45 ^B^	0.001
	0.5 mL/kg	77.94 ± 7.22 ^A^	66.28 ± 9.79 ^B^	66.33 ± 6.43 ^B^	65.67 ± 10.63 ^B^	0.000
	1 mL/kg	77.11 ± 6.98 ^A^	67.11 ± 13.16 ^B^	60.83 ± 6.92 ^B^	67.94 ± 9.83 ^B^	0.000
	*p*_dose_	0.508	0.947	0.096	0.202	*p*_DxG_ = 0.127

Data were presented as mean ± SD. The experimental groups were the control group, the 0.5 mL/kg *Myrtus communis* L. extract, and the 1 mL/kg *Myrtus communis* L. extract. Each individual lamb was considered an independent biological replicate; *n* = 9, 18, and 18 for the control, 0.5 mL/kg, and 1 mL/kg groups, respectively. Different uppercase letters indicate differences among sampling days within the same treatment group (*p* < 0.05). *p*Day, effect of sampling day; *p*Dose, effect of dose group; *p*Dose × Day, dose × day interaction. BUN, blood urea nitrogen; eGFR, estimated glomerular filtration rate; SD, standard deviation.

**Table 5:** Serum ALP, ALT, AST, GGT, and total protein values.

Parameter	Dose	Day 0	Day 15	Day 30	Day 45	*p*_DAY_
ALP (U/L)	Control	292.6 ± 67	237.6 ± 81.9	251.6 ± 48.9	207.3 ± 65.1	0.075
	0.5 mL/kg	346 ± 94.4 ^A^	242.3 ± 75.8 ^B^	282.1 ± 75.9 ^AB^	217.8 ± 52.9 ^B^	0.000
	1 mL/kg	313.7 ± 86.8 ^A^	247.7 ± 68.1 ^AB^	275.3 ± 110.5 ^AB^	234.2 ± 71.3 ^B^	0.036
	*p*_dose_	0.286	0.942	0.694	0.545	*p*_DxG_ = 0.535
ALT (U/L)	Control	24.63 ± 18.7 ^ab^	49.01 ± 35.09 ^a^	27.02 ± 17.19	33.1 ± 23.19	0.575
	0.5 mL/kg	18.15 ± 5.75 ^B,b^	20.86 ± 6.4 ^B,b^	30.09 ± 16.37 ^B^	46.62 ± 30.2 ^A^	0.000
	1 mL/kg	46.82 ± 38.58 ^a^	21.79 ± 7.36 ^b^	52 ± 46.1	78.7 ± 84	0.270
	*p*_dose_	0.006	0.001	0.072	0.109	*p*_DxG_ = 0.749
AST (U/L)	Control	142.6 ± 104.2	158.2 ± 61.7	171.1 ± 53	200.6 ± 85	0.462
	0.5 mL/kg	100.91 ± 23.86 ^C^	136.5 ± 65.8 ^BC^	167.3 ± 68 ^B^	239.5 ± 110.3 ^A^	0.000
	1 mL/kg	174 ± 119.8	204.4 ± 104.9	225.4 ± 123.5	272.8 ± 221.5	0.255
	*p*_dose_	0.062	0.057	0.143	0.545	*p*_DxG_ = 0.581
GGT (U/L)	Control	94.9 ± 32.1	91.52 ± 25.65	94.49 ± 19.45	98.6 ± 19.45	0.945
	0.5 mL/kg	85.76 ± 31.4	83.37 ± 25.62	93.2 ± 34.88	97 ± 27.51	0.496
	1 mL/kg	82.35 ± 18.84	83.13 ± 16.85	85.61 ± 17.57	96.04 ± 16.69	0.080
	*p*_dose_	0.532	0.618	0.603	0.961	*p*_DxG_ = 0.701
Total protein (g/dL)	Control	6.15 ± 0.46 ^B^	6.54 ± 0.36 ^AB^	6.45 ± 0.37 ^AB^	6.82 ± 0.63 ^A^	0.039
	0.5 mL/kg	6.07 ± 0.53 ^B^	6.33 ± 0.41 ^AB^	6.23 ± 0.34 ^AB^	6.52 ± 0.42 ^A^	0.020
	1 mL/kg	6.27 ± 0.49 ^B^	6.35 ± 0.4 ^AB^	6.19 ± 0.49 ^B^	6.68 ± 0.33 ^A^	0.006
	*p*_dose_	0.469	0.393	0.303	0.220	*p*_DxG_ = 0.278

Data were presented as mean ± SD. The experimental groups were the control group, the 0.5 mL/kg *Myrtus communis* L. extract, and the 1 mL/kg *Myrtus communis* L. extract. Each individual lamb was considered an independent biological replicate; *n* = 9, 18, and 18 for the control, 0.5 mL/kg, and 1 mL/kg groups, respectively. Different lowercase letters indicate differences among treatment groups within the same sampling day, whereas different uppercase letters indicate differences among sampling days within the same treatment group (*p* < 0.05). *p*Day, effect of sampling day; *p*Dose, effect of dose group; *p*Dose × Day, dose × day interaction. ALP, alkaline phosphatase; ALT, alanine aminotransferase; AST, aspartate aminotransferase; GGT, gamma-glutamyl transferase; SD, standard deviation. When both uppercase and lowercase letters are shown for the same value, they refer, respectively, to within-group temporal comparisons and between-group comparisons.

**Table 6:** Serum HDL, LDL, total cholesterol, and triglyceride values.

Parameter	Dose	Day 0	Day 15	Day 30	Day 45	*p*_DAY_
Total cholesterol (mg/dL)	Control	35.04 ± 7.92	32.84 ± 4.76	36.13 ± 5.46	37.29 ± 6.4	0.490
	0.5 mL/kg	35.39 ± 13.29	30.42 ± 11.78	31.68 ± 9.06	56.6 ± 97	0.356
	1 mL/kg	35.59 ± 7.43	34.12 ± 8.08	34.98 ± 6.37	35.27 ± 5.81	0.931
	*p*_dose_	0.991	0.490	0.259	0.551	*p*_DxG_ = 0.611
Triglyceride (mg/dL)	Control	19.91 ± 7.55 ^AB^	13.45 ± 5.2 ^B,ab^	15.19 ± 5.31 ^AB^	23.94 ± 9.09 ^A^	0.014
	0.5 mL/kg	19.95 ± 7.78 ^AB^	11.79 ± 3.31 ^C,b^	15.17 ± 4.67 ^BC^	20.64 ± 6.09 ^A^	0.000
	1 mL/kg	19.48 ± 4.79 ^B^	16.53 ± 6.03 ^B,a^	16.62 ± 3.55 ^B^	24.82 ± 4.34 ^A^	0.000
	*p*_dose_	0.975	0.021	0.563	0.125	*p*_DxG_ = 0.292
LDL (mg/dL)	Control	12.69 ± 3	12.16 ± 2.34	14.36 ± 3.39	14.15 ± 3.71	0.382
	0.5 mL/kg	13.61 ± 6.98	11.83 ± 5.75	11.59 ± 4.17	12.61 ± 4.32	0.681
	1 mL/kg	14.29 ± 4.05	14.05 ± 4.36	14.35 ± 3.27	13.93 ± 2.69	0.984
	*p*_dose_	0.757	0.341	0.059	0.453	*p*_DxG_ = 0.255
HDL (mg/dL)	Control	21.13 ± 5.5	20.01 ± 3.51	20.34 ± 2.99	21.3 ± 2.61	0.870
	0.5 mL/kg	20.7 ± 6.51	18.53 ± 6.46	18.33 ± 5.64	20.37 ± 5.15	0.522
	1 mL/kg	19.91 ± 3.76	20.02 ± 4.1	19.01 ± 3.75	20.05 ± 3.95	0.830
	*p*_dose_	0.833	0.637	0.552	0.774	*p*_DxG_ = 0.636

Data were presented as mean ± SD. The experimental groups were control, 0.5 mL/kg *Myrtus communis* L. extract, and 1 mL/kg *Myrtus communis* L. extract. Each individual lamb was considered an independent biological replicate; *n* = 9, 18, and 18 for the control, 0.5 mL/kg, and 1 mL/kg groups, respectively. Different lowercase letters indicate differences among treatment groups within the same sampling day, whereas different uppercase letters indicate differences among sampling days within the same treatment group (*p* < 0.05). *p*Day, effect of sampling day; *p*Dose, effect of dose group; *p*Dose × Day, dose × day interaction. HDL, high-density lipoprotein cholesterol; LDL, low-density lipoprotein cholesterol; SD, standard deviation.

## Data Availability

The raw data supporting the conclusions of this article will be made available by the authors upon request.
